# Patterns of Hamstring Muscle Tears in the General Population: A Systematic Review

**DOI:** 10.1371/journal.pone.0152855

**Published:** 2016-05-04

**Authors:** Barbara Kuske, David F. Hamilton, Sam B. Pattle, A. Hamish R. W. Simpson

**Affiliations:** 1 Department of General Medicine, NHS Lothian, Edinburgh, United Kingdom; 2 Department of Trauma and Orthopaedics, University of Edinburgh, Edinburgh, United Kingdom; Van Andel Institute, UNITED STATES

## Abstract

**Background:**

Hamstring tears are well recognised in the sporting population. Little is known about these injuries in the general population.

**Purpose:**

Evaluating the rates, patterns and risk factors of non-sporting hamstring tears, compared to sporting related hamstring tears.

**Data Sources:**

MEDLINE, EMBASE, CINAHL, and the Cochrane Central Register of Controlled Trials (1989–2015).

**Study Selection:**

Studies reporting patients with a grade 2 or 3 hamstring muscle tear, identified clinically, confirmed by MRI imaging or direct visualisation during surgical exploration.

**Data Synthesis:**

144 sets of linked data were extracted for analysis. Most injuries were in males (81.3%), where mean age at injury was lower (30.2, 95% CI 29.1–31.3) than in females (35.4, 95% CI 32.4–38.4) p = 0.06. Key differences were found in the proportion of non-sporting injuries in patients under and over the age 40 (p = 0.001). The proportion of non-sporting injuries was significantly higher in females compared to males (25.9% female non-sporting injuries, versus 8.5% male; p = 0.02). Avulsions were more frequently reported in non-sporting activities (70.5%). The proportion of such injuries was notably higher in females, though this failed to meet significance (p = 0.124). Grouped by age category a bimodal distribution was noted, with the proportion of avulsions greater in younger (age <15) and older patients (age > 40) (p = 0.008). 86.8% of patients returned to pre-injury activity levels with a similar frequency across all study variables; age, activity (sporting vs non-sporting) and injury type (avulsion vs tear).

**Conclusion:**

This review highlights a proportion of adults suffering grade 2 or 3 hamstring injuries from activities other than the classic sports trauma. The majority of these non-sporting injuries were avulsion injuries that clustered in older female and skeletally immature patients suggesting a potential link to bone mineral density.

## Introduction

Hamstring tears are commonly reported in professional sports players.[1–3] In contrast, the prevalence of hamstring tears in recreational-sporting and non-sporting situations is poorly defined. This lack of data is especially true for older individuals, who are known amongst clinicians to present to general practice and orthopaedic clinics with hamstring tears, yet their prevalence is unknown. Patients suffering a hamstring tear due to sport are mainly in the second and third decade of life.[4, 5] In contrast, the age range of patients with non-sporting or recreational injuries is much greater with hamstring tears reported in patients aged 60 years and over.[1, 6, 7]

A muscle strain is an anatomical and functional disruption of the muscle-tendon unit.[8] Severity of injury is based on the extent of tissue damage, inflammatory response and loss of function. The most common classification system defines a grade 1 injury as no appreciable tissue disruption, mild pain and swelling and no or minimal loss of function, Grade 2 injuries as an identifiable partial disruption of the tissue with moderate pain and swelling thereby leading to loss of function, and grade 3 as complete disruption or tear of the musculotendonous unit with severe pain and swelling and lack of function.[9]

Reports describing hamstring injury diagnosis and management highlight considerable time differences from injury to presentation from several hours to many years.[1, 10] Typical findings on examination include localised tenderness, swelling and a palpable defect. This marked heterogeneity in presenting signs and symptoms along with the sometimes insidious development may lead to delayed presentation and late diagnosis. Diagnosis is aided by imaging, either by plain X-ray to detect bony avulsion fractures or with MRI and ultrasound, which can detail the extent of the injury.[11–14]

Despite their high prevalence, there is a remarkable lack of consensus on hamstring injury management. Recent systematic reviews by Ropiak et al and Ahmad et al suggest that Grade 1 and Grade 2 injuries are typically treated non-operatively.[11, 15] This may encompass rest, ice, compression and progressive mobilization as well as the use of analgesia and non-steroidal anti-inflammatories. The outcome of these partial tears is considered to be varied and unpredictable. Grade 3 injuries and those presenting following failed conservative treatment are often repaired surgically. Good functional outcomes have been reported with operative repair.[1, 16, 17] Correct identification and treatment of suspected hamstring injuries is therefore of high importance.

The aim of this study was to determine the incidence of the injury in both professional and recreational sports people as well as in the remainder of the population, who do not play sport. In particular, our aim was to identify any differences in the type of injury between these groups.

## Methods

### Literature search method

Four major databases were systematically searched in January and February 2015: MEDLINE, EMBASE, CINAHL and the COCHRANE LIBRARY. The complete electronic search history is detailled in S1 Table. Additionally, reference lists of eligible publications for further articles were manually scanned. The search was time limited from January 1989 to January 2015. Two reviewers (BK and SBP) independently read title and abstract of publications identified by the searches potentially meeting the eligibility criteria. Both reviewers read those publications considered relevant in full text to verify they met the eligibility criteria. A third reviewer (DFH) was consulted where initial agreement was not reached.

### Study eligibility criteria

We focused our review on grade 2 and 3 hamstring injuries. To be included, the details of the hamstring injury (distal versus proximal, muscles involved, injury type) had to be stated and confirmed by either imaging (CT, MRI) or by visualisation through surgical exploration, or both. Hamstring avulsion fractures were included, and treatment modality recorded (surgery or conservative management). We required patient age and injury mechanism to be stated. The publication language was English. All data was reviewed including retrospective and case study literature so long as the previous criteria was met.

**Fig 1:**
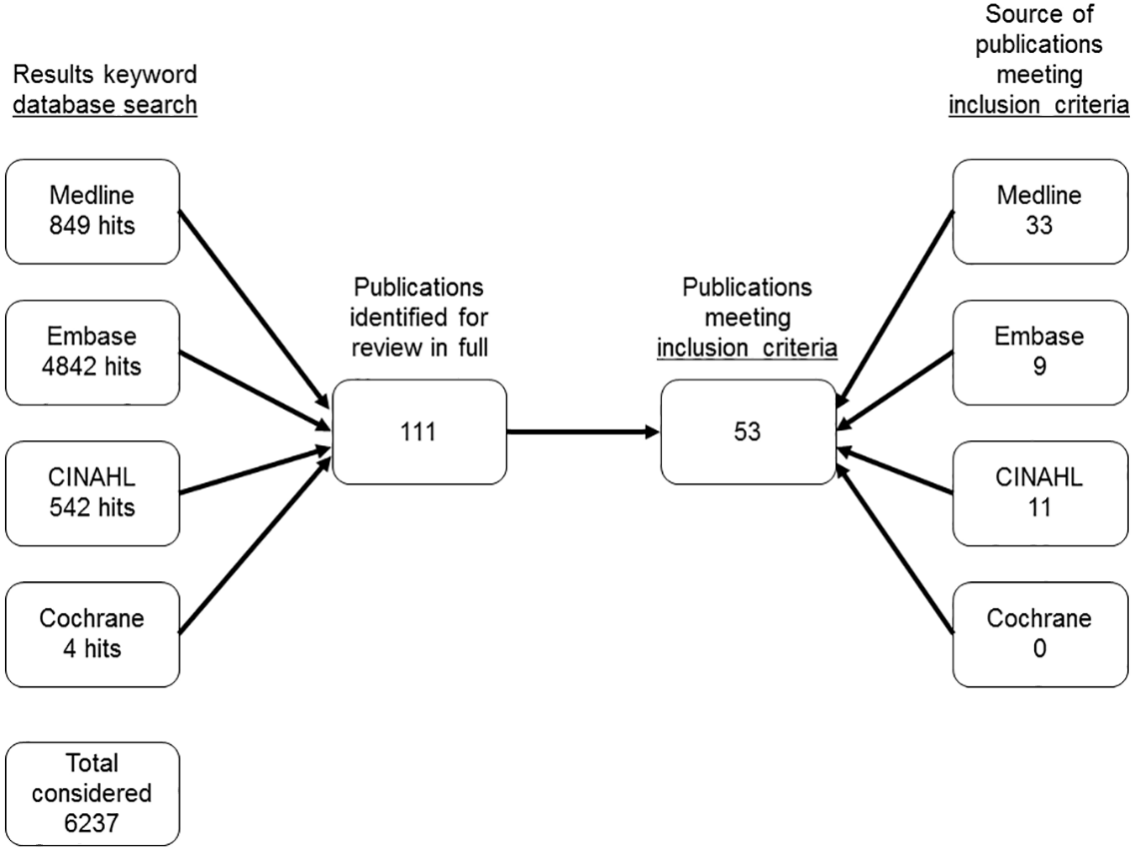
Study Selection Process.

The search resulted in a total of 6237 papers: 849 from MEDLINE (via Ovid 23/01/15), 4842 from EMBASE (via Ovid 1/02/15), 4 from the COCHRANE LIBRARY (1/02/15) and 542 from CINAHL (via EbcsoHost 1/02/15). Following exclusions, 53 papers met the inclusion criteria leading to a final data set of 144 hamstring injuries. The study selection process is shown in Fig 1. The majority of studies included were individual case reports (44) with a smaller number of retrospective case series (2) and a single prospective case series (Table 1).

**Table 1:** Publications summary chart.

Authors	Year	Study design	Number of subjects	non-sporting injury	Subject age (years)
Adejuwon A, et al.[18]	2009	Case series	2	0	19, 25
Alioto RJ, et al.[19]	1997	Case report	1	0	25
Akova B, et al.[20]	2002	Case report	1	0	17
Al-Humadi M, et al.[21]	2009	Case report	1	1	30
Askling C, et al.[22]	2000	Case series	2	0	20, 22
Bansal R, et al.[23]	2005	Case report	1	0	19
Biedert RM [24]	2015	Case Series	3	0	13, 14, 15
Boŝnjak R.[25]	2009	Case report	1	0	48
Bruckner PU, et al.[26]	2005	Case series	8	1*	23–60 (44*)
Carmody C, et al.[27]	1995	Case report	1	0	44
Clark B, et al.[28]	2011	Case report	1	0	26
Cross MJ, et al.[29]	1998	Retrospective review	9	1	54
Cooper D, et al.[5]	2010	Case series	17	0	22–39
Cotofana S, et al.[30]	2012	Case report	1	1	49
Croisier JL, et al.[31]	2005	Case reports	2	0	33, 47
David A, et al.[32]	1994	Case report	1	0	41
Floor S, et al.[33]	2012	Case reports	2	0	44,45
Fortems Y, et al.[34]	1995	Case reports	2	1*	44 (42*)
Gidwani S, et al.[35]	2007	Retrospective review	11	0	9–51
Grieve R, et al.[36]	2006	Case report	1	0	26
Hernesman SC, et al.[6]	2003	Case report	1	0	68
Johnson AE, et al.[37]	2003	Case report	1	1	36
Khoshnoodi P, et al. [38]	2014	Case report	1	0	26
Kirkland A, et al.[39]	2008	Case report	1	0	24
Konan S, et al.[4]	2010	Prospective review	10	0	24–38
Kurosawa H, et al.[40]	1996	Case reports	2	0	25, 28
Kusma M, et al.[41]	2007	Case report	1	0	43
Kwak HY, et al.[42]	2011	Case reports	2	0	16, 24
Kwong Y, et al.[43]	2006	Case report	1	1	62
LaBan MM, et al. [7]	2007	Case report	1	1	64
Lempainen L, et al.[10]	2007	Case series	18	0	18–40
Lin YT, et al. [44]	2008	Case report	1	0	21
Lindner D, et al. [45]	2014	Case report	1	0	16
Mallo G, et al.[46]	2009	Case report	1	0	29
Marx RG, et al.[47]	2009	Case reports	2	1*	30, 30*
Mascarenhas R, et al.[48]	2009	Case report	1	1	22
McGoldrick F, et al.[49]	1990	Case report	1	0	36
Mica L, et al.[50]	2009	Retrospective review	6	6*	(48–73)*
Murray PJ, et al.[51]	2009	Case report	1	0	56
Oseto MC, et al.[52]	2004	Case report	1	0	39
Pan KL, et al. [53]	2000	Case report	1	1	33
Schache AG, et al.[54]	2008	Case report	1	1	20
Scilia A, et al.[55]	2011	Case report	1	0	55
Sebastianelli WJ, et al.[56]	1990	Case report	1	0	21
Servant CTJ, et al. [57]	1998	Case report	1	0	16
Shyamalan G, et al.[58]	2010	Case series	8	2*	11–34 (22*,41*)
Sikka RS, et al. [59]	2013	Case series	3	0	12, 16, 16
Sonnery-Cottet B, et al.[60]	2012	Case report	1	0	24
Stradley SL, et al.[61]	2008	Case report	1	0	43
Takami H, et al. [62]	2000	Case report	1	0	27
Thomson NOB, et al. [63]	1999	Case report	1	0	39
Valente M, et al. [64]	2013	Case report	1	0	24
Watura C, et al.[65]	2011	Case report	1	0	42

*highlight the age of the non-sporting cases in that report.

### Statistical analysis

Data from eligible studies and subjects was collated and analysed using SPSS version 19. Data was reported by mean with 95% confidence intervals as a measure of dispersion. Pearson’s Chi Square, Fisher’s Exact Tests and the Student’s t-test were used to determine association between variables.

## Results

**Fig 2:**
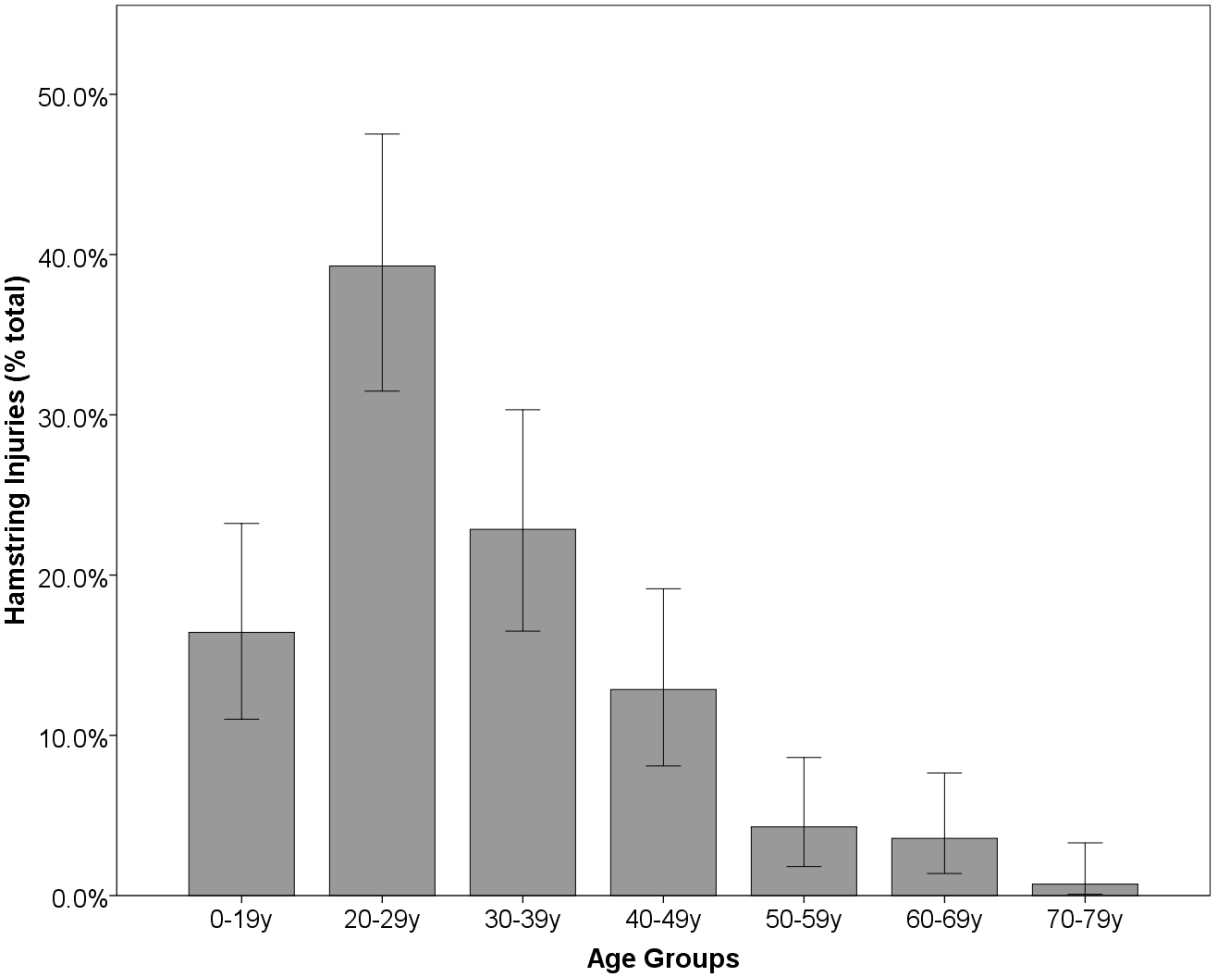
Distribution of reported injuries by age group.

The mean age of patients across the study population was 31.1 (95% CI 30.1–32.3). Patient age ranged from 9 to 73 years. The majority (72.2%) of hamstring injuries reported were observed in patients in their 20s and 30s (Fig 2). Most injuries were reported in males (81.3%). Mean age at injury in males was lower (30.2, 95% CI 29.1–31.324) than in females (35.4, 95% CI 32.4–38.4) with this difference approaching significance (Students t-test, p = 0.06).

### Injurious activity

88.2% of hamstring injuries were described as being sustained during sporting activities (50% of these were in professional or competitive athletes). The most common sporting activities were ball sports (44.4%), running (24.3%), skiing/water skiing (13.2%) and contact sports/martial arts(6.3%). The remaining 11.8% of hamstring injuries were sustained during 'non-sporting' activities; these ranged from a single high impact trauma, road traffic accident, (0.7%) to low impact falls slips and trips (11.1%).

**Fig 3:**
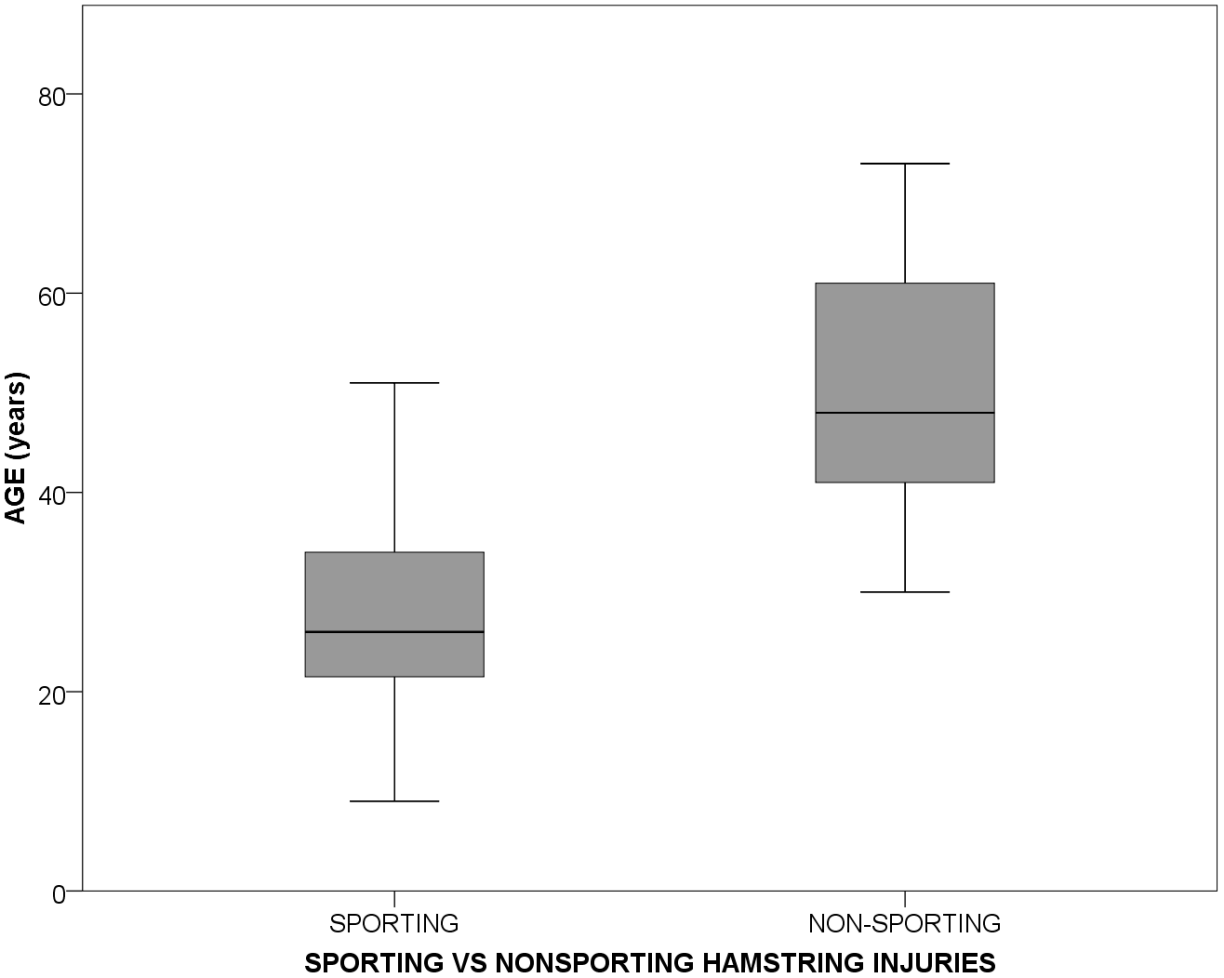
Distribution of injuries comparing sports versus non-sports aetiologies and age.

The mean age of patients with a hamstring injury as a result of playing sport was 28.7 (95% CI 27.7–29.7), compared to a mean age of 49.5 (95% CI 43.0–56.1) in patients with a non-sporting hamstring injury. This difference was statistically significant (p<0.001, Students t-test, Fig 3).

Gender was related to both activity of injury and injury type. The proportion of non-sporting injuries was significantly higher in females compared to males (25.9% of female injuries were non-sporting, compared to 8.5% of male injuries; p = 0.02).

### Injury type

**Fig 4:**
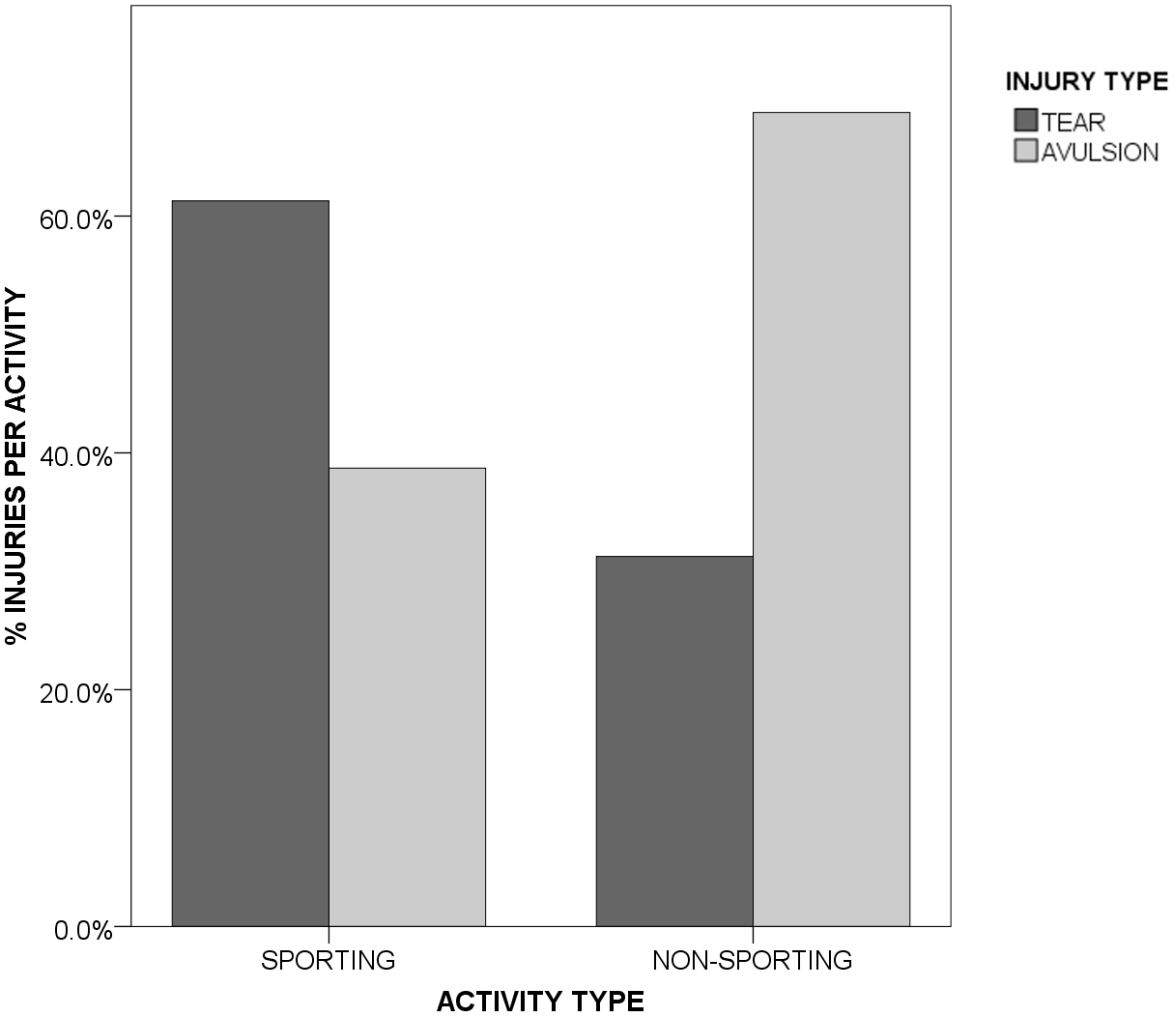
Distribution of injury type by sports participation.

Tears (of muscle or tendon) were the most common injury report (57.6%) the remaining 42.4% being avulsions (of tendon origin/insertion to bone). Avulsion injuries were significantly more common in patients injured during non-sporting activities (70.5%) compared to sports related injuries (38.6%, Fig 4)(p = 0.012, Pearson’s Chi-square test).

The proportion of avulsion injuries was notably higher in females (55.6% of injuries in females were avulsions, compared to 39.3% of injuries in males) compared to males, though this failed to meet significance (p = 0.124, Pearson’s Chi-square test). The mean age was similar across tear and avulsion injury groups (tear30.1y 95% CI 28.1–32.1; avulsion 32.0y 95% CI 29.9–34.1). However, when grouped by age category, the distribution of avulsion injuries was apparently bimodal, being significantly higher in patients below the age of 15 and in patients above the age of 40, compared to those between ages 15 and 39 years (Pearson Chi-Square, p = 0.008).

### Outcomes

Reporting on return to activity was not available for four patients; these patients were excluded from analysis assessing return to pre-injury activity level (the remaining 140 patients were included). 86.8% of patients were described as returning to pre-injury activity levels. There was no difference in age between those who did (30.8yrs, 95%CI 29.6–32.0) and those who did not (30.1yrs, 95%CI 27.5–32.7) return to pre-injury function (Student's t-test, p = 0.798).

## Discussion

Hamstring tears typically occur during rapid acceleration/deceleration involving sudden forced hip flexion concomitant with knee extension, and are well documented to occur as a result of sporting activities. [1, 16] Many studies have assessed the incidence, risk factors and severity of hamstring injuries in professional athletes, perhaps in whom there is a strong economic interest in defining the injury and expediting player return. [66, 67] Our review takes a different approach, assessing all reported hamstring injuries with defined mode of injury including those caused by non-sporting activities. By doing so the patient cohort extends across a much large range.

The principle findings of this systematic review of linked data was a population of individuals injuring their hamstrings in non-sporting situations. These ‘non-sporting injuries’ were more likely in females, in an older demographic and tended to be avulsions of the hamstring origins/insertions to the bone as opposed to tears of the muscle belly

As is typically reported, most hamstring injuries were due to sporting activities, however we highlight a proportion of injuries caused by simple low impact falls and slips; This has not been well described previously. We identified one report looking at non-athletes that described six patients where flexion of the hip with hyperextension of the knee during everyday activities such as missing a step during walking or gardening led to complete hamstring avulsions. [50]

The mean age of people sustaining a non-sporting injury was significantly greater than the age of those suffering sporting injuries. This is in keeping with the current literature, however, the heavily skewed demographic split towards females and the proportion of muscle injury/ avulsion injuries differing between sporting and non-sporting groups are new findings. It is of interest that the avulsions cluster in younger and older individuals, which is consistent witha possible association with skeletal maturity and bone mineral density (BMD). This association between BMD and avulsion/mid substance tendon tear has been previously recognized in different injury sites, namely the calcaneus/Achilles tendon and tibial tubercle/patella tendon; Wren et al demonstrated in a cadaver model that tendo-achilles avulsion injuries were related to reduced calcaneal bone density [68], a finding confirmed in osteoporotic bone and diabetic patients. [69–71] These are most commonly reported in the older patient and are typically the result of minor trauma such as accidental trips causing falls.[72] Similarly, the site where the extensor mechanism of the knee fails varies with age [73], with avulsions occurring at the tibial tubercle/inferior pole of the patella in immature patients and at the superior pole of the patella in older adults.

We suggest that the relatively small number of hamstring injuries reported in non-sporting situations may be a feature of selection bias and an under-representation of the true picture across the general population, as has been observed in other conditions [74]. This is compounded by problems with mis-diagnosis and coding. Hamstring injuries most commonly present with buttock pain, however the differential diagnosis of buttock pain is extensive and includes pathologies in the back, hip and groin. Referred pain from the lumbo-sacral spine may present as ill-defined posterior thigh or buttock pain and is a common presentation in general practice.[11, 75, 76] and may be confused with hamstring tears. In contrast, athletic and surgical cohorts are relatively well coded, in contrast coding of hamstring injuries in general practice is not well defined, again suggesting that hamstring tears in the community may be comparatively under-reported. In the sports medicine literature it is suggested that failing to diagnose a hamstring tear may lead to mismanagement, which may have a detrimental impact on outcome. Although surgical repair of chronic tears can be successful, the procedure is more complex, potentially requiring neurolysis of the sciatic nerve and the outcome is less predictable; consequently early repair is generally recommended [16, 77]. This though is in relation to athletes. The available literature does not confirm whether this same principle applies to the wider population.

### Strengths and Weaknesses

The main limitation of this analysis of linked data was the inability to proceed to formal meta-analysis. This was due to the overall poor quality of study variable definition in the individual reports.

However, despite this limitation, it has identified a population of hamstring tears due to non-sporting injuries, particularly in females, which should be considered in the differential diagnosis of hip pain following a low energy fall. Further research is needed in this group to ascertain the most appropriate intervention and management especially in those where there is an avulsion fracture.

## Conclusion

This review highlights a largely unrecognised population of adults suffering hamstring injuries from low-impact activities. These tend to be avulsion injuries that cluster in the older female and skeletally immature patients suggesting a potential link to bone mineral density.

## Supporting Information

S1 PRISMA Flow Diagram. (DOCX)

S2 PRISMA Research Checklist. (DOCX)

S1 Table Search strategy. (DOCX)
