# Supplementary material for: Patterns of Hamstring Muscle Tears in the General Population: A Systematic Review
Source: PLoS One. 2016 May 4;11(5):e0152855. doi: 10.1371/journal.pone.0152855 (PMC4856270; doi:10.1371/journal.pone.0152855)
Supplement: S1 PRISMA — (DOCX) [file pone.0152855.s001.docx]

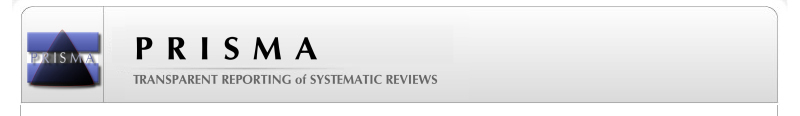
**PRISMA 2009 Flow Diagram**

Studies included in quantitative synthesis (meta-analysis)
(n = )

Studies included in qualitative synthesis
(n =53 )

Full-text articles excluded, with reasons
(n = 58 )

Full-text articles assessed for eligibility
(n = 111 )

Records excluded
(n =5595 )

Records screened
(n = 642 )

Records after duplicates removed
(n = ) –not done

Additional records identified through other sources
(n = 0 )

## Identification

## Eligibility

## Included

## Screening

Records identified through database searching
(n = 6237 )
