# Supplementary material for: Patterns of Hamstring Muscle Tears in the General Population: A Systematic Review
Source: PLoS One. 2016 May 4;11(5):e0152855. doi: 10.1371/journal.pone.0152855 (PMC4856270; doi:10.1371/journal.pone.0152855)
Supplement: S1 Table — (DOCX) [file pone.0152855.s003.docx]

S1 Table: Search Strategy

| **Search strategy for Medline (via Ovid SP)**  Search executed 23/01/2015  Resources: Ovid Medline(R) 1946 to Present with Daily Update and Ovid Medline(R) In-Process & Other Non-Indexed Citations  Advancedsearch  Keyword   1. expTendonInjuries/ 2. expAthleticInjuries/ 3. exp "SprainsandStrains"/ 4. ((hamstring$ or semimembran$ or semitend$ or biceps$ femoris$) adj3 (injur$ or tear$ or avulsion$ or rupture$)).tw. 5. hamstring.tw. 6. leg injury.tw. 7. 1 or 3 or 2 8. 6 or 4 or 5 9. 7 and 8 10. Limit 9 to year = ‘1989-current’ |
| --- |
| **Search strategy for EMBASE (via Ovid SP)**  Search executed 01/02/2015  Resources: EMBASE  Advanced search   1. hamstring.tw. 2. leg injury.tw. 3. ((hamstring$ or semimembran$ or semitend$ or biceps$ femoris) adj3 (injur$ or tear$ or avulsion$ or rupture$)).tw. 4. 1 or 3 or 2 5. Limit 4 to year = ‘1989-current’ |
| **Search strategy for CINAHL PLUS (EBSCOhost)**  Search executed 1/02/2015  Database: CINAHL PLUS with Full Text  Advancedsearch   1. TX tendon injuries or TX athletic injuries or TX (sprains and strains) 2. TX hamstring 3. S1 and S2 4. Limited by publication type: -academic journal 5. Limited bypublisheddate 19890101-20130916 |
| **Search strategy for Cochrane Library (direct search via Wiley Online Library)**  Search executed 16/09/2013  Title, abstracts, keyword: “Hamstring”  Limit publication year from 1989 to 2015 |
